# Racial disparities in alpha‐fetoprotein testing and alpha‐fetoprotein status associated with the diagnosis and outcome of hepatocellular carcinoma patients

**DOI:** 10.1002/cam4.2549

**Published:** 2019-09-13

**Authors:** Guoyi Wu, Jing Wu, Xiaoben Pan, Bo Liu, Zhicheng Yao, Yuan Guo, Xiaolei Shi, Yitao Ding

**Affiliations:** ^1^ Department of Hepatobiliary Surgery The Affiliated Drum Tower Hospital of Nanjing University Medical School Nanjing Jiangsu China; ^2^ Clinical Medical Center for Digestive Disease of Jiangsu Province Nanjing Jiangsu China; ^3^ National Center for Chronic and Noncommunicable Disease Control and Prevention Chinese Center for Disease Control and Prevention Beijing China; ^4^ Hangzhou Key Laboratory of Inflammation and Immunoregulation Department of Basic Medical Science School of Medicine Hangzhou Normal University Hangzhou Zhejiang China; ^5^ Department of General Surgery The Third Affiliated Hospital of Sun Yat‐sen University Guangzhou Guangdong China; ^6^ Department of Infectious Diseases The Second Affiliated Hospital of Chongqing Medical University Chongqing China

**Keywords:** alpha‐fetoprotein, diagnosis, end results, epidemiology, hepatocellular carcinoma, overall survival, racial disparities, surveillance

## Abstract

**Background:**

The use of alpha‐fetoprotein (AFP) testing for the surveillance, diagnosis, and prognosis of hepatocellular carcinoma (HCC) remains controversial. Here, we compared AFP testing rates, elevated AFP rates, factors associated with elevated AFP levels, and prognostic factors associated with overall survival (OS) in HCC patients from different ethnic groups.

**Methods:**

Patients with HCC were identified from the Surveillance, Epidemiology, and End Results registries. Race was categorized as white, black, and others. AFP testing rates and elevated AFP rates were analyzed. Multivariable logistic regression and Cox regression analyses were used to identify independent factors associated with elevated AFP levels and prognosis, respectively. All statistical tests were two sided.

**Results:**

A proportion of 79.2% of total HCC patients had AFP testing reports; 77.3% of white, 79.7% of black, and 81.2% of other races underwent AFP testing. Compared with white and other races, black HCC patients had a higher rate of elevated AFP levels among all patients and the early‐stage HCC patient cohort. Elevated AFP level was a significant prognostic factor for all HCC patients in different race groups. Factors associated with elevated AFP level and prognostic factors associated with OS varied significantly by race.

**Conclusions:**

AFP testing, elevated AFP rates, predictors of elevated AFP level, and prognostic factors associated with OS differed significantly according to race after adjusting for AFP levels among the three groups. AFP testing for the surveillance, diagnosis, and prognosis of HCC patients is strongly recommended, although racial disparities need to be considered.

## Graphical abstract

We hypothesized that the racial disparities of the elevated AFP in HCC patients may be an important issue regarding to the difference of using AFP for HCC surveillance and diagnosis between Asian and the United States HCC patients. Our results showed that elevated AFP rates were high in all three race groups, and the highest rate of AFP tests performed and elevated was the black race group, which was reported to be the least likely to present with an elevated AFP at diagnosis. Our present findings suggest that AFP testing could still be considered for the diagnosis, and prognosis of HCC patients with more attention to the black race.

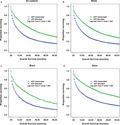

## 1 INTRODUCTION

Hepatocellular carcinoma (HCC) is one of the most common cancers and the fourth leading cause of cancer‐related mortality worldwide.^1^ The incidence rate of HCC shows the fastest increase among all cancers in the United States, and the number of HCC cases have doubled in the last decade likely because of increased obesity/diabetes.^2^ HCC mortality rates are increasing in the United States, although the burden of HCC is not equally distributed among different races.^3^

Biannual screening of high‐risk populations via ultrasonography is not widely adopted by physicians in the United States, despite the fact that early detection improves the prognosis of HCC patients.^4^, ^5^ The rationale for HCC screening in patients with high‐risk factors (cirrhosis and hepatitis) is that screening tests such as ultrasonography or serum alpha‐fetoprotein (AFP) measurement may help identify HCC patients at an early stage, at which the disease is potentially curative or has life‐prolonging treatment options, including local destruction, surgical resection, or liver transplantation.^6^ Curative treatments are only available for HCC patients diagnosed at an early stage, and the prognosis of HCC depends on tumor stage at diagnosis.^7^ Early‐stage HCC patients have 5‐year survival rates approaching 70% after surgical resection or liver transplantation, compared with a median survival of 1 year for those at advanced stages.^8^

The incidence and mortality rates of HCC vary according to race/ethnicity, which is mainly attributed to differences in the prevalence of major risk factors and disparities in access to high‐quality care.^9^ Black race HCC patients are more likely to be diagnosed at advanced stages, less likely to follow the recommended treatment regimens, and more like to have worse survival outcomes compared with white race patients.^10^ So, it is more important to perform the screening tests in high‐risk black populations. Racial differences in the levels of AFP as a diagnostic marker for HCC have been investigated in different studies, and opposite results were reported. Some researchers consider that AFP is not accurate for the diagnosis of HCC in African Americans,^11^ whereas other groups reported that African Americans show increased levels of AFP.^12^ These conclusions were made based on data from multicenter clinical studies that included hundreds of patients with hepatitis C. However, to the best of our knowledge, no population‐based studies have been performed to date.

In this report, we examined the rates of AFP testing performed and the proportions of elevated AFP levels in HCC patients using a United States population‐based database, and reported contemporary overall survival (OS) rates for three different racial groups.

## 2 METHODS

### 2.1 Surveillance, Epidemiology, and End Results (SEER) data and selection of patients

SEER program data are maintained by the United States National Cancer Institute, including information on cancer incidence, pathology, and survival for approximately 28% of the US population. All data were obtained from the publicly available SEER database using SEER*Stat 8.3.5 (NCI). Patients presenting with HCC between 2004 and 2015 were identified using the International Classification of Diseases for Oncology, Third Edition (ICD‐O‐3) topography and morphology codes from SEER 18 Regs Research Data. Our preliminary sample included patients with “primary site” code “C22.0” and “histologic type” codes “8170 to 8175”. Among 68 473 HCC cases identified, patients were excluded in a stepwise fashion if they had unknown age or age < 18 years old (n = 138), unknown race (n = 280), unknown AJCC TNM stage of cancer (n = 12 173), as well as autopsy only or death certificate only (n = 681) cases, leaving a final analysis sample of 55 201 HCC cases. Patients with available AFP testing records were selected for further analyses of the factors associated with elevated AFP levels at HCC diagnosis and survival. The dataset used is publicly available, and approval by the institutional review board at Nanjing Drum Tower Hospital (Nanjing, Jiangsu, China) was waived.

### 2.2 Independent variables

The SEER database code data on the AFP level at diagnosis as “Positive/elevated,” “Negative/normal,” “Borderline; undetermined whether positive or negative,” and other undocumented situations. HCC patients with AFP data classified as “Positive/elevated,” “Negative/normal,” and “Borderline; undetermined whether positive or negative,” were identified as “AFP testing performed”. The SEER program uses the physician's determination of AFP levels reported in the medical record when available or the reference values provided by the laboratory reporting the result. We grouped HCC according to race as “whites,” “blacks,” and “others” (American Indians/Alaska Natives, and Asian/Pacific Islanders). Age was divided into quintiles (18‐44 years vs 45‐54 years vs 55‐64 years vs 65‐74 years vs >75 years); year of diagnosis was divided into quartiles (2004‐2006 vs 2007‐2009 vs 2010‐2012 vs 2013‐2015); and the stage of HCC was grouped into quartiles (stage I vs stage II vs stage III vs stage IV) following the 6th edition of the AJCC Cancer Staging Manual. The type of treatment was defined as “surgery performed,” “Not recommended,” and “Others.”

### 2.3 Statistical analysis

Descriptive statistics were reported as medians with interquartile range for continuous variables and as whole numbers and percentages for categorical variables. Descriptive statistics for discrete variables were compared using the Chi square test. Factors associated with AFP testing were identified using multivariable logistic regression analysis. The Kaplan‐Meier method was used to assess OS stratified by race and APF level. A multivariable Cox proportional‐hazards model was built to determine the prognostic factors of OS. The prognostic power of covariates was expressed as hazard ratio (HR) with 95% confidence interval (CI). Statistical significance was defined as *P* < .01 (two sided). All analyses were performed using SPSS version 23.0 (IBM).

## 3 RESULTS

### 3.1 Patient characteristics

A total of 55 201 patients with HCC who were diagnosed between 2004 and 2015 and who met the study inclusion criteria were identified, including 33 789 whites (68.5%), 7628 blacks (13.8%), and 9784 others (17.7%). More than three quarters of patients (76.9%) were male. The median age of patients with HCC was 62 years (interquartile range, 56‐71 years) and varied according to race (63 years for whites, 56 years for blacks, and 56 years for others). The number of HCC diagnosed increased from 9492 cases in the 2004‐2006 period to 17 844 cases in 2013‐2015. More than 79.2% of patients had AFP testing records. Other demographic and clinical characteristics of the cohort were shown in Table 1.

**Table 1:** Descriptive characteristics of 55 201 cases of HCC using the SEER data from 2004 to 2015

Variable	Frequency	%
Total	55 201	100.0
Race		
White	37 789	68.5
Black	7628	13.8
Others	9784	17.7
Sex		
Male	42 433	76.9
Female	12 768	23.1
Age, y		
18‐44	1640	3.0
45‐54	9198	16.7
55‐64	20 826	37.7
65‐74	13 502	24.5
75 up	10 035	18.2
Year of diagnosis		
2004‐2006	9492	17.2
2007‐2009	12 465	22.6
2010‐2012	15 400	27.9
2013‐2015	17 844	32.3
AFP record		
Performed	43 695	79.2
Not performed	11 506	20.8
AJCC Stage		
I	21 635	39.2
II	11 019	20.0
III	13 103	23.7
IV	9444	17.1
Grade		
Well differentiated	6554	11.9
Moderately	9298	16.8
Poorly	4410	8.0
Undifferentiated	386	0.7
Unknown	34 553	62.6
Fibrosis score		
0‐4	2963	5.4
5‐6	12 160	22.0
Not applicable/unknown	40 078	72.6
Treatment		
Surgery performed	14 978	27.1
Not recommended	37 796	68.5
Others	2427	4.4

Abbreviation: AFP, Alpha‐fetoprotein; HCC, Hepatocellular Carcinoma; SEER, Surveillance, Epidemiology, and End Results.

### 3.2 AFP testing disparities stratified by race and diagnostic period

**Figure 1:**
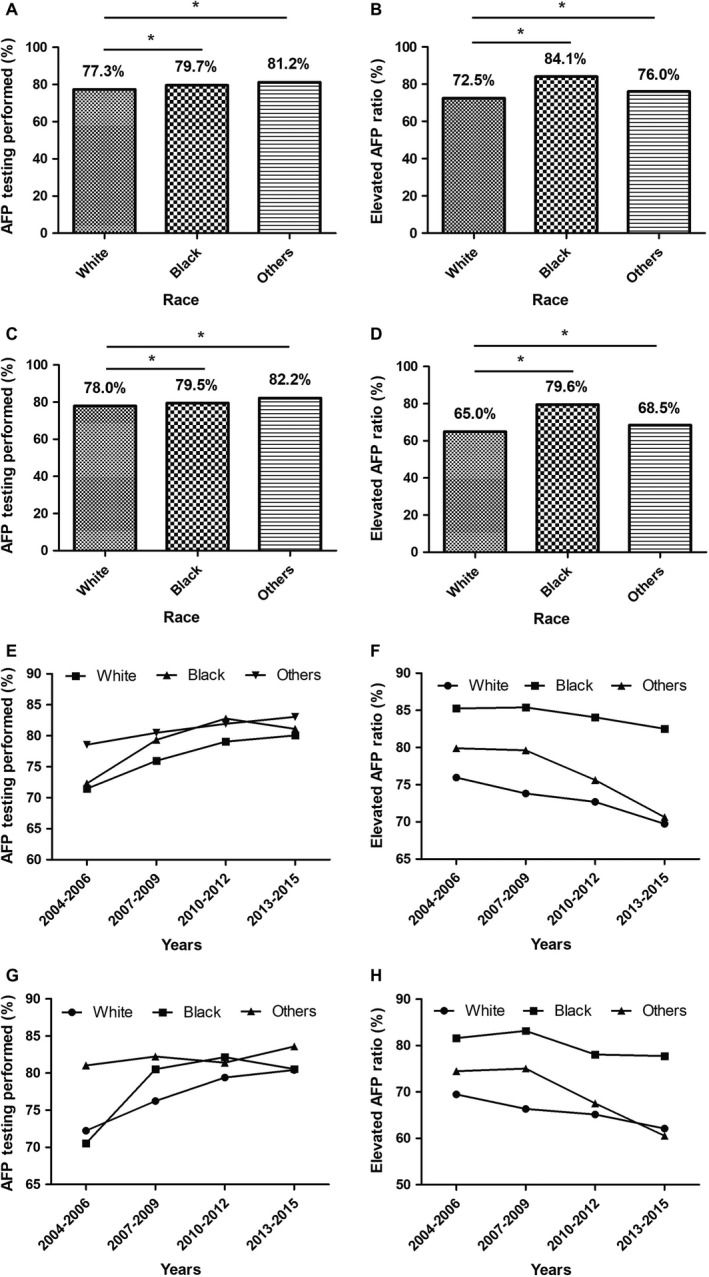
Ratios of AFP testing performed and elevated AFP level by race. A, Performing rates of AFP testing among HCC patients by race. B, Elevated AFP level proportion among HCC patients by race. C, Performing rates of AFP testing among early‐stage HCC patients by race. D, Elevated AFP level proportion among early‐stage HCC patients by race. E, Trends of AFP testing performing rates among HCC patients by race. F, Trends of elevated AFP level proportion among HCC patients by races. G, Trends of AFP testing performing rates among early‐stage HCC patients by races. H, Trends of elevated AFP level proportion among early‐stage HCC patients by race. *P < .01

The overall proportion of patients who underwent AFP testing was higher in black (79.7%) and other race (81.2%) patients than in white patients (77.3%) (ure 1A). Among patients with available AFP testing records, black patients had the highest rate (84.1%) of elevated AFP, whereas white patients had the lowest rate (72.5%) of elevated AFP (Figure 1B). In the subset cohort of HCC patients diagnosed at an early stage (AJCC stage I), the proportion of patients who underwent AFP testing remained higher in the black (79.5%) and other race (82.2%) groups than in the white patients group (78.0%) (Figure 1C). Meanwhile, the rates of elevated AFP decreased in all three race groups, but black patients still presented the highest rate (79.6%) among them (Figure 1D).

The period trend of patients who underwent AFP testing increased from 71.5% in 2004‐2006 to 80.1% in 2013‐2015 in white patients, and from 72.3% to 81.1% in black patients (Figure 1E) at the same period in the whole cohort. The proportion of elevated AFP decreased from 76.0% to 69.7% in white patients, and from 85.3% to 82.5% in black patients, as determined by comparing the 2004‐2006 period to the 2013‐2015 period (Figure 1F). The period trends of the performing rates of AFP testing and elevated AFP rates in the subcohort of early‐stage HCC patients were shown in Figure 1G,H, respectively.

### 3.3 Factors associated with elevated AFP levels according to race

Factors associated with increased AFP levels according to race were analyzed in the cohort with available AFP testing results. Multivariate analysis (Table 2) showed that female white [vs male white; odds ratio (OR), 1.18; 95% CI, 1.11‐1.26; *P* < .01] and female other race HCC patients (vs male other race; OR, 1.50; 95% CI, 1.33‐1.70; *P* < .01) were associated with a significantly higher OR of elevated AFP at diagnosis. However, among black race patients, gender was not associated with elevated AFP level. In the white HCC patient group, age 45‐54 years (vs age 18‐44 years; OR, 2.14; 95% CI, 1.79‐2.55; *P* < .01), age 55‐64 years (vs age 18‐44 years; OR, 2.01; 95% CI, 1.47‐2.23; *P* < .01), age 65‐–74 years (vs age 18‐44 years; OR, 1.51; 95% CI, 1.27‐1.79; *P* < .01), age > 75 years (vs age 18‐45 years; OR, 1.43; 95% CI, 1.21‐1.71; *P* < .01) were significantly associated with elevated AFP level. In patients older than 45 years, younger white HCC patients were more likely to be AFP positive. However, the association between age and elevated AFP level was not significant in the black and other race groups. Multivariate analysis showed that higher AJCC stage, lower tumor grade, and higher liver fibrosis score were associated with a higher incidence of increased AFP levels in these three race groups, although the ORs were different among them. Detailed results were shown in Table 2.

**Table 2:** Multivariate analyses of the mediators associated with elevated AFP level by race

Variable	White		Black		Other	
	OR (95% CI)	*P* Value	OR (95% CI)	*P* Value	OR (95% CI)	*P* Value
Sex						
Male	Ref		Ref		Ref	
Female	1.18 (1.11‐1.26)	.00	NA	NA	1.50 (1.33‐1.70)	.00
Age (years)						
18‐44	Ref		Ref		Ref	
45‐54	2.14 (1.79‐2.55)	.00	1.42 (0.95‐2.13)	.09	0.84 (0.62‐1.10)	.19
55‐64	2.01 (1.69‐2.38)	.00	1.21 (0.83‐1.76)	.32	0.81 (0.61‐1.05)	.11
65‐74	1.51 (1.27‐1.79)	.00	0.91 (0.62‐1.33)	.61	0.68 (0.52‐0.90)	.01
75 up	1.43 (1.21‐1.71)	.00	0.80 (0.52‐1.21)	.29	0.61 (0.46‐0.81)	.00
AJCC Stage						
I	Ref		Ref		Ref	
II	1.49 (1.39‐1.59)	.00	1.59 (1.31‐1.92)	.00	1.51 (1.32‐1.73)	.00
III	2.28 (2.11‐2.46)	.00	1.80 (1.49‐2.18)	0.00	2.37 (2.04‐2.76)	.00
IV	2.21 (1.21‐1.71)	.00	1.79 (1.43‐2.24)	.00	2.80 (2.30‐3.42)	.00
Grade						
Well differentiated	Ref		Ref		Ref	
Moderately	1.60 (1.45‐1.75)	.00	1.59 (1.27‐2.01)	.00	1.46 (1.22‐1.76)	.00
Poorly	2.68 (2.36‐3.05)	.00	2.94 (2.12‐4.08)	.00	2.33 (1.85‐2.95)	.00
Undifferentiated	2.37 (1.63‐3.44)	.00	1.90 (0.72‐4.97)	.19	2.97 (1.51‐5.81)	.00
Unknown	2.24 (2.07‐2.42)	.00	2.34 (1.93‐2.83)	.00	2.33 (1.98‐2.74)	.00
Fibrosis score						
0‐4	Ref		Ref		Ref	
5‐6	1.28 (1.13‐1.45)	.00	1.68 (1.25‐2.25)	.001	1.35 (1.11‐1.64)	.00
Not applicable/ unknown	1.22 (1.08‐1.37)	.00	1.46 (1.12‐1.90)	.01	1.42 (1.20‐1.70)	.00
Tumor Size (cm)						
≤2.0	Ref		Ref		Ref	
2.1‐5.0	1.35 (1.25‐1.46)	.00	1.32 (1.06‐1.64)	.01	1.08 (0.92‐1.28)	.34
5.1‐10.0	1.74 (1.59‐1.91)	.00	1.48 (1.12‐1.89)	.00	1.26 (1.05‐1.52)	.01
≥10.1	1.83 (1.63‐2.05)	.00	1.40 (1.06‐1.86)	.02	1.45 (1.17‐1.80)	.00
Unknown	2.25 (1.99‐2.54)	.00	1.88 (1.38‐2.55)	.00	1.68 (1.27‐2.23)	.00

Abbreviation: AFP, Alpha‐fetoprotein.

### 3.4 Elevated AFP as a prognostic factor of OS stratified by race

**Figure 2:**
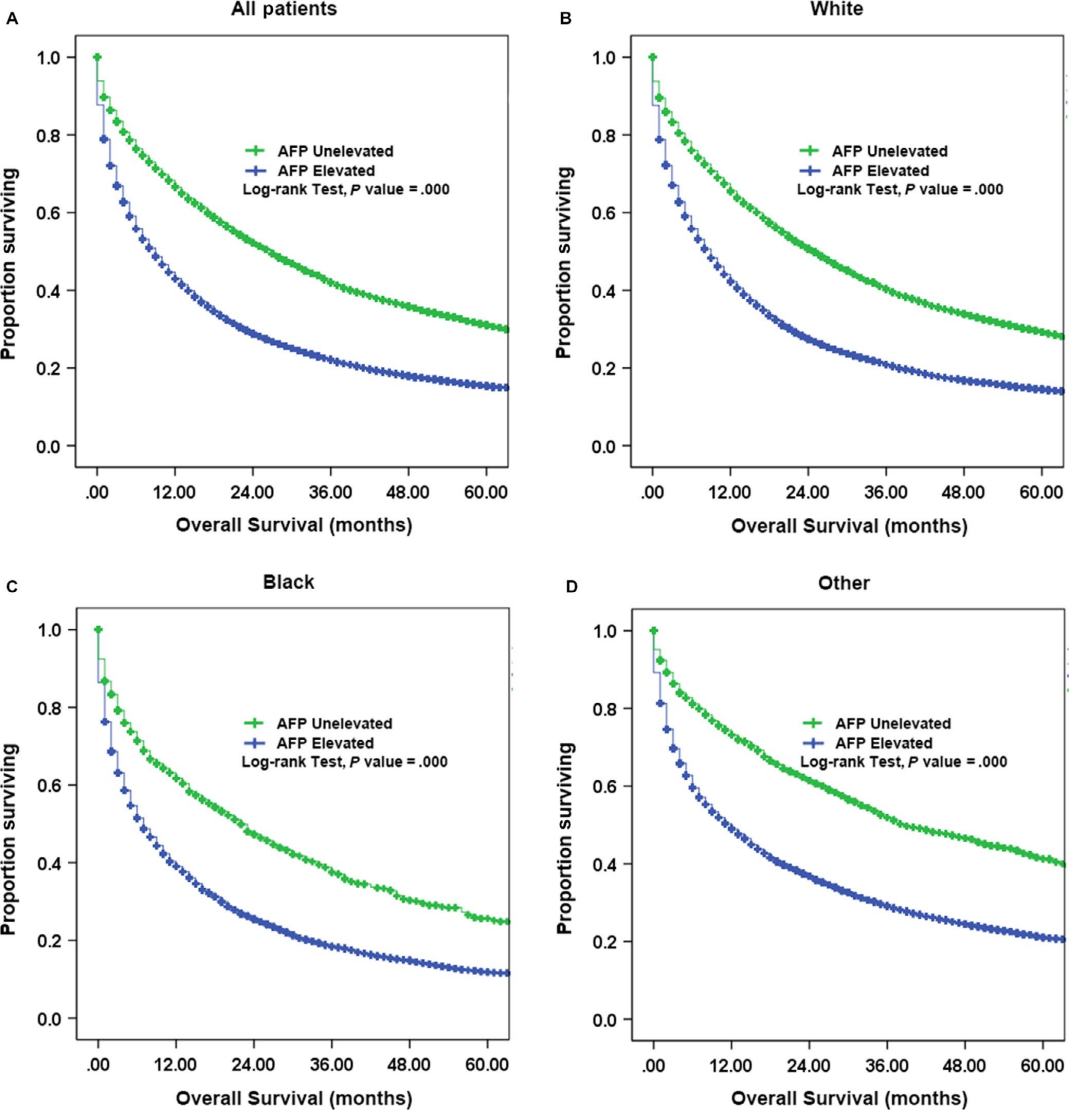
Kaplan‐Meier curves for OS of HCC patients by AFP level. A, OS in all patients with unelevated or elevated AFP level. B, OS in white race patients with unelevated or elevated AFP level. C, OS in black race patients with unelevated or elevated AFP level. D, OS in other race patients with unelevated or elevated AFP level

The median OS for the entire cohort with AFP records and survival information was 11 months; the 1‐, 3‐, and 5‐year survival rates were 66.5%, 42.0%, and 31.1%, respectively. AFP‐positive patients had a lower median OS (9 months) than those with normal AFP levels (27 months). After stratification by race, the median OS was worse in the AFP‐elevated patients than in the AFP nonelevated patients in each race groups, with the values of 7 vs 22 months in the black race group, 9 vs 25 months in the white race group, and 12 vs 39 months in the other race group, respectively (Figure 2).

On multivariable analysis, elevated AFP levels were associated with worse OS in each race group (white: HR, 1.41, 95% CI 1.37‐1.46, *P* < .01; black: HR, 1.46, 95% CI 1.34‐1.59, *P* < .01; and other race: HR, 1.46, 95% CI 1.36‐1.56, *P* < .01) (Table 3). However, other prognostic factors were different among the three races after adjusting for AFP levels. Female gender was a significant prognostic factor for better outcomes in the black race group (HR 0.85; 95% CI 0.79‐0.91; *P* < .01), whereas it was not significant in the white race group (HR 0.96; 95% CI 0.93‐0.99; *P* = .01) or other race group (HR NA; 95% CI NA; *P* = .40). White patients aged 45‐54 years ( HR, 1.29; 95% CI, 1.17‐1.43; *P* < .01), 55‐64 years (HR, 1.28; 95% CI, 1.16‐1.41; *P* < .01), and >75 years (HR, 1.74; 95% CI, 1.57‐1.93; *P* < .01) were associated with a worse OS compared with 18‐44‐year‐old patients. The association of age with poor OS was not significant among black patients in the 45‐65‐year‐old group or in other race patients aged 45‐75 years compared with younger patients (18‐44 years) in the respective groups. Notably, the higher tumor stages are significantly associated with the worse OS in different race group (Stage IV vs stage I: white: HR, 2.50, 95% CI 2.40‐2.61, *P* < .01; black: HR, 2.22, 95% CI 2.02‐2.43, *P* < .01; and other race: HR, 3.26, 95% CI 2.98‐3.58, *P* < .01), respectively. Although poorly tumor grades were associated with the worse OS, however, moderately tumor grades were not associated with the poor OS in each race group (Table 3). Other prognostic factors were shown in Table 3.

**Table 3:** Multivariate cox regression of prognostic factors of HCC patients by race

Variable	White		Black		Other	
	HR (95% CI)	*P* Value	HR (95% CI)	*P* Value	HR (95% CI)	*P* Value
AFP						
Negative	Ref		Ref		Ref	
Positive	1.41 (1.37‐1.46)	.00	1.46 (1.34‐1.59)	.00	1.46 (1.36‐1.56)	.00
Sex						
Male	Ref		Ref		Ref	
Female	0.96 (0.93‐0.99)	.01	0.85 (0.79‐0.91)	.00	NA	NA
Age (years)						
18‐44	Ref		Ref		Ref	
45‐54	1.29 (1.17‐1.43)	.00	1.33 (1.13‐1.57)	.00	0.97 (0.86‐1.13)	.83
55‐64	1.28 (1.16‐1.41)	.00	1.27 (1.09‐1.49)	.00	0.97 (0.85‐1.09)	.59
65‐74	1.45 (1.31‐1.61)	.00	1.37 (1.17‐1.62)	.00	1.04 (0.92‐1.18)	.56
75 up	1.74 (1.57‐1.93)	.00	1.67 (1.39‐2.00)	.00	1.27 (1.12‐1.44)	.00
AJCC Stage						
I	Ref		Ref		Ref	
II	1.10 (1.05‐1.14)	.00	1.17 (1.07‐1.28)	.00	1.13 (1.04‐1.23)	.00
III	1.67 (1.61‐1.74)	.00	1.56 (1.44‐1.69)	.00	1.99 (1.85‐2.15)	.00
IV	2.50 (2.40‐2.61)	.00	2.22 (2.02‐2.43)	.00	3.26 (2.98‐3.58)	.00
Grade						
Well differentiated	Ref		Ref		Ref	
Moderately	1.06 (1.00‐1.18)	.04	1.16 (1.03‐1.31)	.02	0.96 (0.85‐1.08)	.46
Poorly	1.45 (1.36‐1.54)	.00	1.54 (1.35‐1.76)	.00	1.23 (1.08‐1.39)	.00
Undifferentiated	1.31 (1.11‐1.55)	.00	2.20 (1.57‐3.08)	.00	1.55 (1.16‐2.08)	.00
Unknown	1.289 (1.23‐1.35)	.00	1.39 (1.26‐1.54)	.00	1.15 (1.05‐1.27)	.00
Fibrosis score						
0‐4	Ref		Ref		Ref	
5‐6	1.14 (1.06‐1.23)	.00	0.99 (0.85‐1.15)	.86	1.10 (0.97‐1.24)	.13
Not applicable/ unknown	1.32 (1.23‐1.42)	.00	1.23 (1.07‐1.41)	.00	1.27 (1.14‐1.41)	.00
Tumor size (cm)						
≤2.0	Ref		Ref		Ref	
2.1‐5.0	1.34 (1.28‐1.41)	.00	1.42 (1.26‐1.60)	.00	1.45 (1.29‐1.62)	.00
5.1‐10.0	1.85 (1.75‐1.96)	.00	1.78 (1.57‐2.01)	.00	1.97 (1.76‐2.22)	.00
≥10.1	2.34 (2.20‐2.49)	.00	2.49 (2.17‐2.85)	.00	3.00 (2.65‐3.40)	.00
Unknown	2.77 (2.60‐2.95)	.00	3.14 (2.73‐3.60)	.00	3.07 (2.67‐3.52)	.00
Therapy						
Surgery performed	Ref		Ref		Ref	
Recommended	2.42 (2.26‐2.60)	.00	2.15 (1.86‐2.49)	.00	1.99 (1.71‐2.33)	.00
Not Recommended	2.52 (2.42‐2.62)	.00	2.45 (2.24‐2.68)	.00	2.39 (2.22‐2.27)	.00
Unknown	3.00 (2.82‐3.20)	.00	2.76 (2.42‐3.15)	.00	3.04 (2.65‐3.48)	.00

Abbreviation: AFP, Alpha‐fetoprotein; HCC, Hepatocellular Carcinoma.

## 4 DISCUSSION

Serum AFP level is not considered as the best indicator for HCC according to some guidelines.^13^, ^14^, ^15^ This is due to the low sensitivity and high false‐negative rate of serum AFP for the diagnosis of HCC, as reported in recent studies.^1^, ^16^ In addition, AFP testing is suboptimal because of its cost in routine surveillance of early‐stage HCC.^17^ However, the Liver Cancer Study Group of Japan 15 and Guidelines for Diagnosis and Treatment of Primary Liver Cancer in China 18 still recommend the opposite approach, supporting that the main strategies for early screening of HCC are serum AFP testing in combination with liver ultrasonography.

To the best of our knowledge, this is the first population‐based study assessing racial disparities among patients undergoing AFP testing and the rates of elevated AFP among patients with HCC in the United States. The present study analyzed a large cohort of patients diagnosed with HCC, and showed significant disparities in the rate of AFP testing performed and elevated AFP rate among three race groups. The results showed that black (79.7%) and other race (81.2%) HCC patients were more likely to undergo AFP testing than white race patients (77.3%). Moreover, significant differences in the incidence of elevated AFP were analyzed according to race, with a significantly higher rate of elevated AFP in black patients than in white patients. A previous study of racial disparities in AFP testing showed that blacks accounted for approximately half (43%) of the patients with HCC and a normal AFP level.^11^ Our findings were consistent with the results of the HALT‐C trial, which showed that elevated AFP levels were more frequent in blacks.^12^ The differences in the results could be due to the variations in methodology, such as patient selection (the previous studies included HCC patients with hepatitis C and both were multicenter based clinical studies), or they could represent differences among patients at different stages of HCC. To clarify this issue, we analyzed the disparities in AFP testing and elevated AFP levels in early‐stage HCC patients. After controlling for the stage of HCC, the AFP testing performing rate in black patients was still higher than that of white patients. The analysis of the early‐stage HCC cohort revealed a decrease in the proportion of elevated AFP in each race group, however, black race patients remained the group with the highest rate of undergoing AFP testing and proportion of elevated AFP. Our results also indicated that regardless of racial differences, AFP testing performing rates increased, and the positive AFP rates declined in each HCC patients race group during the 2004‐2015 period.

Despite the present findings that the black HCC patient group was more likely to have a higher proportion of patients with elevated AFP levels, other disparities associated with elevated AFP levels were observed among the three race groups. Female white HCC patients aged 45‐64 years, with late AJCC stage, and with larger tumor size were more likely to have elevated AFP levels. However, gender was not a factor associated with elevated AFP levels, and age was not significantly associated with elevated AFP levels in black HCC patients. Unlike the white or black race group, the older patients (≥65 years) in other race group were less likely to have elevated AFP levels. Therefore, disparities in the rate of elevated AFP among HCC patients need to be further analyzed after stratification by race.

Although the use of AFP testing for the early diagnosis of HCC is controversial, serum AFP is recommended as a prognostic biomarker for HCC outcome in clinical practice.^19^, ^20^, ^21^, ^22^ However, the role of AFP levels at diagnosis as an independent risk predictor associated with OS remains unclear.^23^, ^24^ Our data indicate that elevated AFP level is a significant prognostic factor for HCC OS in all three race groups. However, after adjusting for AFP level and other factors, age was not an effective predictor of OS in the other race group, whereas it was a predictor in the white race group. Fibrosis score was not a prognostic factor for OS in black and other race groups after controlled by AFP level. Even after controlling for AFP level and other prognostic factors, disparities in other prognostic factors were still existed among female patients in the other race group.

### 4.1 Limitations

The present study had a strong feature in its generalizability, as the findings were based on population data, but the study had several limitations. First, we did not have data on the actual numerical value of AFP levels. Normal and elevated AFP values, and a uniform cutoff value were lacking, and we had to rely on local interpretation. However, we believe that similar and standardized reference values are used in most laboratories in the United States. Second, there may have been testing, reporting, and interpretation differences in AFP values between hospitals both within and across the SEER cancer registries. It is not clear to what extent the bias introduced by nonrandom missing data and/or variation in the reporting of AFP status would change our results. Additionally, the potential for mis‐classification of race needs to be considered, as the data were extracted from medical records. However, taken together with the comparisons of case proportions and survival by race, our results are robust and informative. The present study provides evidence of racial differences in the use of AFP testing as a biomarker for HCC surveillance, diagnosis, as well as a prognostic factor.

## 5 CONCLUSION

In conclusion, the present study demonstrated that AFP testing is important for the diagnosis and prognosis of HCC in different racial groups. However, racial disparities in the factors associated with elevated AFP levels among the three race groups were existed. We strongly recommend the combination of AFP testing with ultrasonography for the detection of HCC, especially for black race patients. In addition, combining patient demographic characteristics, especially race, with AFP testing would increase the value of AFP testing for HCC surveillance and diagnosis. AFP level remained a prognostic factor for HCC even after accounting for demographics and tumor characteristics; however, further studies are needed to improve the prediction of HCC patient outcomes, especially among racial minorities.

## AUTHOR CONTRIBUTIONS

Drs Guoyi Wu and Jing Wu had full access to all of the data in the study and take responsibility for the integrity of the data and the accuracy of the data analysis. Guoyi Wu, Jing Wu, and Xiaoben Pan were involved in study concept and design, and statistical analysis. Guoyi Wu, Xiaoben Pan, and Bo Liu were involved in acquisition, analysis, or interpretation of data. Guoyi Wu, and Jing Wu were involved in drafting of the manuscript. Zhicheng Yao, Yuan Guo, Xiaolei Shi, and Yitao Ding were involved in critical revision of the manuscript for important intellectual content. Guoyi Wu, Jing Wu, and Yitao Ding obtained funding. Xiaolei Shi, and Yitao Ding were involved in study supervision.

## FUNDING INFORMATION

The National Key Research and Development Program of China (grant no. 2016YFC1302603). Nanjing University Hospital Management Institute, (grant/Award Number: NDYG2016014). The Clinical Medical Center for Digestive Disease of Jiangsu Province (BL2012001).
