# Supplementary material for: Racial disparities in alpha‐fetoprotein testing and alpha‐fetoprotein status associated with the diagnosis and outcome of hepatocellular carcinoma patients
Source: Cancer Med. 2019 Sep 13;8(15):6614–23. doi: 10.1002/cam4.2549 (PMC6825973; doi:10.1002/cam4.2549)
Supplement: Supplementary file 1 [file CAM4-8-6614-s001.doc]

**Table S1.** Univariate Cox Regression of Prognostic Factors of HCC Patients by Race.

|  | **White** | | **Black** | | **Other** | |
| --- | --- | --- | --- | --- | --- | --- |
| **Variable** | HR (95% CI) | P Value | HR (95% CI) | P Value | HR (95% CI) | P Value |
| **AFP** |  |  |  |  |  |  |
| Negative | Ref |  | Ref |  | Ref |  |
| Positive | 1.75 (1.69-1.81) | 0.00 | 1.73 (1.58-1.88) | 0.00 | 1.85 (1.73-1.99) | 0.00 |
| **Sex** |  |  |  |  |  |  |
| Male | Ref |  | Ref |  | Ref |  |
| Female | 0.90 (0.87-0.93) | 0.02 | 0.80 (0.75-0.86) | 0.00 | 0.92 (0.87-0.98) | 0.01 |
| **Age (years)** |  |  |  |  |  |  |
| 18-44 | Ref |  | Ref |  | Ref |  |
| 45-54 | 1.28 (1.15-1.41) | 0.00 | 1.11 (0.94-1.31) | 0.20 | 0.88 (0.77-1.00) | 0.05 |
| 55-64 | 1.24 (1.19-1.36) | 0.00 | 1.04 (0.89-1.22) | 0.60 | 0.84 (0.74-0.95) | 0.01 |
| 65-74 | 1.44 (1.30-1.59) | 0.00 | 1.10 (0.93-1.29) | 0.28 | 0.87 (0.77-0.99) | 0.03 |
| 75 up | 1.95 (1.76-2.16) | 0.00 | 1.40 (1.17-1.68) | 0.00 | 1.19 (1.05-1.35) | 0.01 |
| **AJCC Stage** |  |  |  |  |  |  |
| I | Ref |  | Ref |  | Ref |  |
| II | 1.04 (1.00-1.08) | 0.05 | 1.02 (0.99-1.18) | 0.09 | 1.09 (1.01-1.18) | 0.04 |
| III | 2.59 (2.50-2.69) | 0.00 | 2.39 (2.21-2.57) | 0.00 | 3.17 (2.96-3.40) | 0.00 |
| IV | 4.49 (4.31-4.67) | 0.00 | 3.91 (3.59-4.25) | 0.00 | 6.26 (5.77-6.78) | 0.00 |
| **Grade** |  |  |  |  |  |  |
| Well differentiated | Ref |  | Ref |  | Ref |  |
| Moderately | 1.01 (0.96-1.07) | 0.64 | 1.09 (0.97-1.23) | 0.14 | 0.93 (0.81-1.01) | 0.08 |
| Poorly | 1.85 (1.73-1.97) | 0.00 | 1.86 (1.63-2.12) | 0.00 | 1.45 (1.29-1.65) | 0.00 |
| Undifferentiated | 1.93 (1.63-2.28) | 0.00 | 2.94 (2.10-4.12) | 0.00 | 1.75 (1.31-2.34) | 0.00 |
| Unknown | 1.62 (1.55-1.69) | 0.00 | 1.69 (1.53-1.87) | 0.00 | 1.57 (1.42-1.73) | 0.00 |
| **Fibrosis score** |  |  |  |  |  |  |
| 0-4 | Ref |  | Ref |  | Ref |  |
| 5-6 | 1.17 (1.09-1.26) | 0.00 | 1.12 (0.97-1.30) | 0.00 | 1.16 (1.03-1.30) | 0.02 |
| Not applicable/ unknown | 1.64 (1.53-1.76) | 0.00 | 1.65 (1.44-1.88) | 0.00 | 1.71 (1.54-1.90) | 0.00 |
| **Tumor Size (cm)** |  |  |  |  |  |  |
| ≤2.0 | Ref |  | Ref |  | Ref |  |
| 2.1-5.0 | 1.52 (1.45-1.60) | 0.00 | 1.54 (1.37-1.73) | 0.00 | 1.48 (1.32-1.66) | 0.00 |
| 5.1-10.0 | 2.96 (2.81-3.12) | 0.00 | 2.63 (2.33-2.96) | 0.00 | 3.01 (2.69-3.37) | 0.00 |
| ≥10.1 | 3.82 (3.60-4.05) | 0.00 | 3.92 (3.45-4.46) | 0.00 | 4.88 (4.34-5.49) | 0.00 |
| Unknown | 5.46 (5.14-5.80) | 0.00 | 5.53 (4.85-8.31) | 0.00 | 6.03 (5.28-6.89) | 0.00 |
| **Therapy** |  |  |  |  |  |  |
| Surgery Performed | Ref |  | Ref |  | Ref |  |
| Recommended | 2.94 (2.75-3.15) | 0.00 | 2.72 (2.36-3.13) | 0.00 | 2.43 (2.09-2.84) | 0.00 |
| Not Recommended | 3.61 (3.48-3.74) | 0.00 | 3.61 (3.32-3.92) | 0.00 | 3.55 (3.32-3.79) | 0.00 |
| Unknown | 4.20 (3.25-5.42) | 0.00 | 4.19 (2.62-6.70) | 0.00 | 3.01 (1.25-7.24) | 0.01 |

Abbreviation: AFP, Alpha-fetoprotein; HCC, Hepatocellular Carcinoma.
